# About Face: Matching Unfamiliar Faces Across Rotations of View and Lighting

**DOI:** 10.1177/2041669517744221

**Published:** 2017-11-29

**Authors:** Simone Favelle, Harold Hill, Peter Claes

**Affiliations:** School of Psychology, University of Wollongong, Wollongong, New South Wales, Australia; ESAT/PSI, Department of Electrical Engineering, KU Leuven, Belgium; Medical Imaging Research Center, UZ Leuven, Belgium

**Keywords:** face perception, viewpoint, lighting, unfamiliar face matching

## Abstract

Matching the identities of unfamiliar faces is heavily influenced by variations in their images. Changes to viewpoint and lighting direction during face perception are commonplace across yaw and pitch axes and can result in dramatic image differences. We report two experiments that, for the first time, factorially investigate the combined effects of lighting and view angle on matching performance for unfamiliar faces. The use of three-dimensional head models allowed control of both lighting and viewpoint. We found viewpoint effects in the yaw axis with little to no effect of lighting. However, for rotations about the pitch axis, there were both viewpoint and lighting effects and these interacted where lighting effects were found only for front views and views from below. The pattern of effects was similar regardless of whether view variation occurred as a result of head (Experiment 1) or camera (Experiment 2) suggesting that face matching is not purely image based. Along with face inversion effects in Experiment 1, the results of this study suggest that face perception is based on shape and surface information and draws on implicit knowledge of upright faces and ecological (top) lighting conditions.

## Introduction

The image of a face is not only determined by its shape and reflectance but also by the directions from which it is viewed and lit. Rotations of viewpoint result in occlusion and accretion of facial features and surfaces as well as changes to the shape of the face’s outline. A profile view, for example, has one less eye, eyebrow and cheek compared with a front view, but that view provides information regarding the protuberance of the nose not directly available from a front view. The appearance of a face can change at least as dramatically with changes in lighting direction. Indeed, Adini, Moses, and Ullman (1997) made objective comparisons of face images rendered under various viewing conditions and demonstrated that changes in lighting accounted for greater image variance than changes in viewpoint (and that both accounted for greater image variance than changes in identity). Extrinsic factors such as viewpoint and lighting direction together with the intrinsic three-dimensional (3D) shape and surface reflectance properties of a face determine the pattern of shading and shadows, as well as the surfaces of a face that are visible (Hill & Bruce, 1996; Liu, Collin, & Chaudhuri, 2000). Successfully isolating the intrinsic information that allows us to discriminate identity is a major challenge for any visual system. In this study, we investigate how viewpoint and lighting together impact the processing of this intrinsic, identity-specific face information using a sequential matching task with unfamiliar faces.

While matching faces across changes in images is a challenge (Moses, Ullman, & Edelman, 1996), it is one that the human visual system is generally very good at solving for familiar faces (Hancock, Bruce, & Burton, 2000; Johnston & Edmonds, 2009). Familiar faces appear to be processed in a largely view and lighting-invariant manner, which is most likely a consequence of an observer’s previous experience of the face and the multiple views (and possible variations of those views) stored for those faces (Burton, Jenkins, & Schweinberger, 2011). Unfamiliar face recognition, on the other hand, appears to be more closely tied to low-level image information (Hancock et al., 2000; O’Toole, 2005), leaving it particularly vulnerable to image variation. Many studies have shown that unfamiliar face perception and recognition is viewpoint dependent (Hill, Schyns, & Akamatsu, 1997; Jeffery, Rhodes, & Busey, 2006; Lee, Matsumiya, & Wilson, 2006; Liu, & Chaudhuri, 2002; Newell, Chiroro, & Valentine, 1999; O’Toole, Edelman, & Bülthoff, 1998; O’Toole, Vetter, & Blanz, 1999; Troje & Bülthoff, 1996; Wallraven, Schwaninger, Schuhmacher, & Bülthoff, 2002) as well as lighting dependent (Adini et al., 1997; Braje, 2003; Braje, Kersten, Tarr, & Troje, 1998; Hill & Bruce, 1996; Johnston, Hill, & Carman, 1992; Tarr, Georghiades, & Jackson, 2008; Tarr, Kersten, & Bulthoff, 1998). However, despite both lighting and viewpoint codetermining the image, their combined effect has received considerably less attention. The aim of this article is to investigate that relationship.

Our focus in this study is on cues to identity that are carried by differences in the 3D shape of the face. While it is clear that surface reflectance (including pigmentation) is important for recognition (O’Toole et al., 1999; Russell, Biederman, Nederhouser, & Sinha, 2007), we concentrate on shape because it is the projection of the 3D shape of the face into two-dimensional (2D) images that is primarily affected by lighting and viewing direction. The interaction of viewing angle or lighting direction with the 3D shape of the face determines which surfaces are visible and the 2D projections of those surfaces. The perception of surface properties is based on how an observer *interprets* luminance variations in such images (e.g., as being due to the presence of a contour, shading, surface pigmentation, or shadows) and will affect their ability to recognise faces (Bruce & Langton, 1994; Kemp, Pike, White, & Musselman, 1996; Liu, Collin, Burton, & Chaudhuri, 1999).

Lighting and viewpoint effects in face recognition implicate shape and shading information as playing an important role in the representations that mediate face perception. Hill and Bruce (1996) examined the combined effects of lighting and viewpoint on performance matching 3D face shape. Faces (3D facial surfaces without colour or texture) were rotated 0°, 45° and 90° in depth about the vertical axis (yaw) with either top or bottom lighting. Matching faces across yaw rotations of viewpoint was more accurate with top lighting than bottom lighting and matching faces between changes in top lighting was more accurate than between changes in bottom lighting. Top lighting relative to the observer did not provide a benefit for matching inverted faces, suggesting that changes in viewpoint and lighting have specific effects on face processing, beyond any general effect on pattern matching. Hill and Bruce (1996) argue that their results could not be explained in terms of image or edge differences and were consistent with top lighting assisting in the interpretation of the 3D shape of face stimuli. However, alternative accounts based simply on our greater experience with top-lit faces cannot be ruled out.

While Hill and Bruce (1996) showed that viewpoint effects depend on lighting direction their research was focussed on the effects of lighting and limited to the effects of top or bottom lighting on yaw rotation of faces. There are nontrivial differences between yaw and pitch rotations of faces. While both types of depth rotation result in self-occlusion (e.g., one cheek and half the jaw are largely occluded in yaw rotation and the forehead occluded in pitch views from below), the bilateral symmetry of the face may allow for occluded information in yaw rotations to be more easily recoverable (Troje & Bulthoff, 1996). The same argument could apply to lighting, another reason to compare both view and lighting manipulations across both axes. The aim of this study was to do this: Factorially examine the interaction between the effects of viewpoint and lighting within the yaw and pitch axes of depth rotation. Research has shown that face recognition is viewpoint dependent in each of 3D (yaw, pitch and roll) axes (Favelle, Palmisano, & Avery, 2011; Favelle, Palmisano, & Maloney, 2007; Wallraven et al., 2002). Note, however, that both the overall performance and rate of viewpoint decline depended on the axis of rotation (Favelle et al., 2011). Specifically, performance on a face recognition task was best and declined slowest for roll camera rotations and become poorer with increasingly steeper declines for yaw, pitch-below and pitch-above camera rotations, respectively. Lighting was primarily from above (including ambient lighting) and held constant with respect to the face while the camera viewpoint was rotated. While this lighting was ecologically based, it could be a factor in explaining Favelle et al.’s results. It is possible that the pattern of lighting on the face may have been used an additional cue to recognition and the lighting was especially detrimental to pitch view change compared with roll or yaw views. The findings of Hill and Bruce (1996) and Favelle et al. (2007, 2011) together point to the need to investigate lighting and viewpoint more closely in combination.

Analogies can be drawn between the effects of lighting and viewpoint. For example, rotations of the face (or camera) in pitch are similar in some respects to the effect of changing between top and bottom lighting as both correspond to rotations around the mediolateral or horizontal axis of the head. Likewise, rotations of the face (or camera) in yaw are analogous to changes from left to right side lighting as both involve rotations around the vertical axis of the head. The surfaces visible from a particular viewpoint largely correspond to the surfaces that would receive direct illumination if lit from that direction and occluding contours correspond to attached shadow boundaries. In this study, we examined both top or bottom and left or right lighting conditions with respect to head and camera rotations about both pitch and yaw. We expected that performance would be best with top lighting (Hill & Bruce, 1996; Johnston et al., 1992). However, if surface information is important for successful matching then we would expect to find superior performance in conditions lit from directly in front of the face since the pattern of light remains constant and there are no shadows obscuring the surface. Comparing patterns of performance in left or right versus top or bottom lighting conditions can test the extent to which lighting may be treated as reflectance change. If the effect of lighting is based largely on changes in luminance across the images, then patterns for left or right lighting should be similar to top or bottom. The overall pattern of generalisation will provide information about the relative importance of changes in view and lighting and about the extent to which these depend on each other.

**Figure 1.**
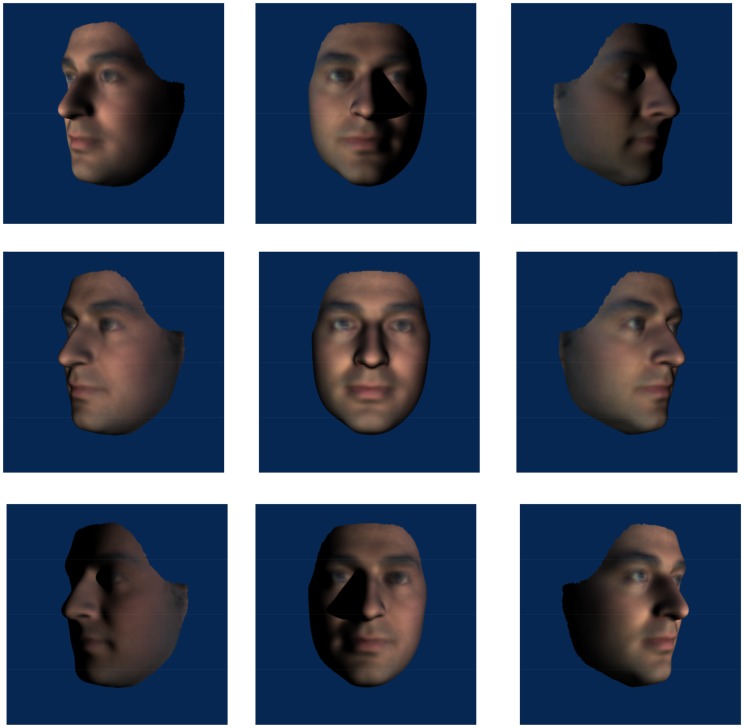
Examples of stimuli in which the *head* was rotated about the yaw axis (left column 45° leftwards, middle column 0° and right column 45° rightwards) and lighting rotated about the yaw axis (top row 45° leftwards, middle row 0° and bottom row 45° rightwards).

**Figure 2.**
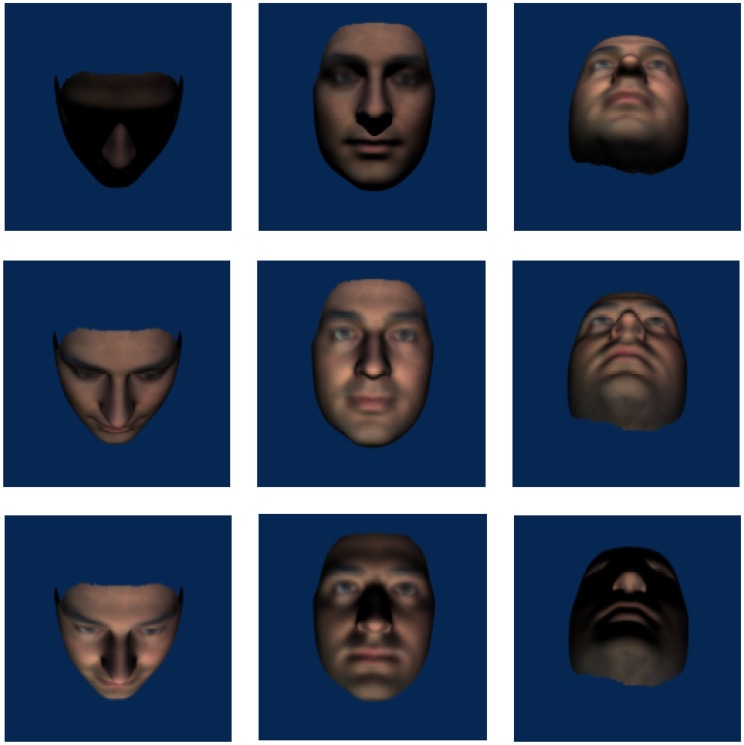
Examples of stimuli in which the *head* was rotated about the pitch axis (left column 45° downwards, middle column 0° and right column 45° upwards) and lighting rotated about the pitch axis (top row 45° top lighting, middle row 0° front lighting and bottom row 45° bottom lighting).

When considering the effect of view rotation with respect to face recognition, there are two ways in which view rotation can occur. First, the observer is in a fixed position and the face or head is rotated about a particular axis (with the origin of this rotation being at the observer). Second, the face or head is in a fixed position and the observer’s viewpoint instead moves or rotates (with the origin of these rotations being the face). While the images produced by both types of movement will have considerable overlap; there will be some important points of difference. During head rotation, the light field will be uniform with respect to the observer but the pattern of light and dark on the surface of the face will change. By contrast, during observer movement, the pattern of light and dark on the surface of the face will remain unchanged (as the light field is uniform with respect to the head). This distinction results in 2D image differences (compare Figures 1 and 6 or Figures 2 and 7) and would be particularly important if the pattern of lighting on the face (as distinct from the pattern in the image) is important for matching or subsequent recognition (e.g., a highlight on a particular facial feature). This difference in the pattern of lighting has not been studied previously despite the potential importance as a cue and variations across studies (i.e., head vs. camera rotation). We test whether patterns of generalisation reflect this, hypothesising that if the pattern of lighting on the surface is important for matching, camera rotations (Experiment 2) will be less detrimental than head rotations (Experiment 1) as the former leaves these patterns unchanged.

Finally, class-based knowledge is likely to be important for face recognition (Adini et al., 1997). It is argued that faces are processed by a specialised face recognition system based on findings of stronger inversion and composite effects, for example, for faces than for objects (McKone, 2010; McKone & Robbins, 2011). Contrast reversal effects have also been found to be greater for faces than objects (Nederhouser, Yue, Mangini, & Biederman, 2007; Vuong, Peissig, Harrison, & Tarr, 2005) and present for identity but not expression judgements (White, 2001). In order to determine whether there is a specialised role for viewpoint and lighting in a dedicated face recognition system, we included inverted conditions in Experiment 1. Russell et al. (2007) showed that face inversion disrupted the use of shape and reflectance about equally. The presence of an inversion effect in this study would suggest that matching across viewpoint and lighting depends on implicit knowledge of upright faces.

## Experiment 1

In Experiment 1, we investigated the effect of head rotation on face matching. That is, the camera remained fixed at a front view and the head and lighting rotated about both pitch and yaw axes.

### Method

#### Participants

Sixty-seven undergraduate psychology students from the University of Wollongong served as participants for this experiment as a part of a course requirement. Ethical approval for all experiments was obtained from the University of Wollongong Human Research Ethics Committee, application HE08/067, in accordance with Australian National guidelines. All participants gave informed consent with an approximate ratio of male to female participants of 1:4. All had normal or corrected-to-normal vision and none were familiar with the facial stimuli used in the experiment.

#### Stimuli

3D models of synthetic faces were used to create the stimuli for all between- and within-subject conditions. This gives ready control over viewpoint and lighting while keeping shape and reflectance constant. A total of 180 synthetic faces were created from a pool of 54 real faces, 27 males and 27 females, recorded using a surface scanning device, NEC “Fiore” (Yip, Smith, & Yoshino, 2004; Yoshino, Matsuda, Kubota, Imaizumi, & Miyasaka, 2000). This uses distortions of patterned light to recover shape and also records images of surface texture. Correspondences between face scans were established using an automatic method where each face was represented in terms of a mesh with 9,327 vertices (Claes, Vandermeulen, De Greef, Willems, & Suetens, 2006). Face models were then rendered with a 3D modelling software package, “Blender” version 2.45 (www.blender.com) using the Internal Raytracing engine to generate 480 × 480 JPEG images. All faces were rendered using an average texture map to model reflectance. Only the face (and not the whole head) was rendered to restrict the available information to the face. This is somewhat analogous to the use of an oval mask used with photographic front views to remove hair and ear cues. The camera was 50 cm from the head and had a modelled 60 mm focal length. Lighting was *sun* type which is directional. *Energy* was 1.5 Blender units and does not fall-off with distance for this type of modelled light. Stimuli were presented on a Dell computer with a 17 in. screen using a program written in Adobe Director.

For Experiment 1, the stimuli were created with the camera remaining fixed (at 0°) with front and three-quarter (45°) head rotations and lighting rotations about both pitch and yaw axes. View angles were combined factorially with lighting angles for each axis such that there were nine different views for each axis (see Figures 1 and 2), including a common front-lit front view of the face. Inverted stimuli were created by rotating the face images 180°, thus, bottom lighting becomes top lighting relative to the observer.

#### Procedure

The experiment took place in a dimly lit room. The experiment began with a set of written instructions and a practice session using different faces shown under the same conditions.

The experiment consisted of 90 randomly ordered trials (10 trials per condition) in each of which subjects were presented with a two alternative forced choice (2AFC) matching-to-sample task. A self-paced break was given halfway through (i.e., after 45 trials). Each trial began with a fixation cross appearing for 250 ms at the centre of the screen followed by a single face presented for 1,500 ms. This face was replaced with a random noise pattern mask appearing on the screen for 200 ms followed by a 300 ms blank screen and then followed by a pair of test faces presented either side of the centre of the screen (no central fixation cross) for 2,000 ms which was also replaced with a random noise pattern mask. The mask remained on the screen until a response was made. Participants were asked to decide which of the two test faces was the same as the learned face, by responding *left* or *right* using the corresponding arrow keys. The next trial began 1,000 ms after the subject made a response. No feedback was given on response accuracy. The single learned face was always presented at light angle 0°, view angle 0°. The two test faces were presented in the same view and lighting angle condition as each other. The faces used in each trial, the side on which the target face appeared, the vertical position of each of the two faces (±50 pixels) and the size of each face (63% or 83% of learned face) was fully randomised. For trials in inverted conditions, both the learned and test faces were presented in the inverted orientation.

#### Design

We used a four-factor design: 2 Orientation (upright and inverted) × 2 Axis of Rotation (yaw and pitch) × 3 Light Angle (−45°, 0°, 45°) × 3 View Angle (−45°, 0°, 45°). The between-subject factors were orientation and axis resulting in four groups. Light angle and view angle were manipulated within subjects. The 2AFC matching-to-sample task involved deciding which of two test faces corresponded to the learned face – target faces and paired distractor faces were chosen randomly for each participant separately from the 180 synthetic faces available without attempting to control for, but avoiding any bias associated with, perceived attributes like sex, age or distinctiveness. There were 10 trials in each condition giving a total of 3 × 3 × 10 = 90 trials for each participant.

### Results

Analyses were conducted on proportion correct face matching data.^1^ Pitch and yaw rotations were analysed separately with 2 Orientation (upright or inverted) × 3 Lighting Angle (−45°, 0°, 45°) × 3 Viewing Angle (−45°, 0°, 45°) mixed-design analysis of variances (ANOVAs). The α level was .05. All post hoc comparisons were Bonferroni-adjusted pairwise comparisons (adjusted *p* values are reported). Where the assumption of sphericity was violated a Greenhouse-Geisser correction was used. The number of participants in each condition was as follows: upright yaw, 16; inverted yaw, 16; upright pitch, 19; and inverted pitch, 16.

#### Yaw rotations

The three-way mixed-design ANOVA showed a main effect of the between-subject factor of orientation, *F*(1, 30) = 14.32, *p* < .001, $\eta_{p}^{2}$ = .32. Matching accuracy was significantly greater for upright (*M* = .83, *SD* = .08) than inverted (*M* = .72, *SD* = .08) faces. There was also a significant Light × View interaction, *F*(4, 120) = 5.83, *p* < .001, $\eta_{p}^{2}$ = .16, and main effect of View, *F*(2, 60) = 14.91, *p* < .001, $\eta_{p}^{2}$ = .33. None of the other main effects or interactions was significant (all *p*’s > .1).

**Figure 3.**
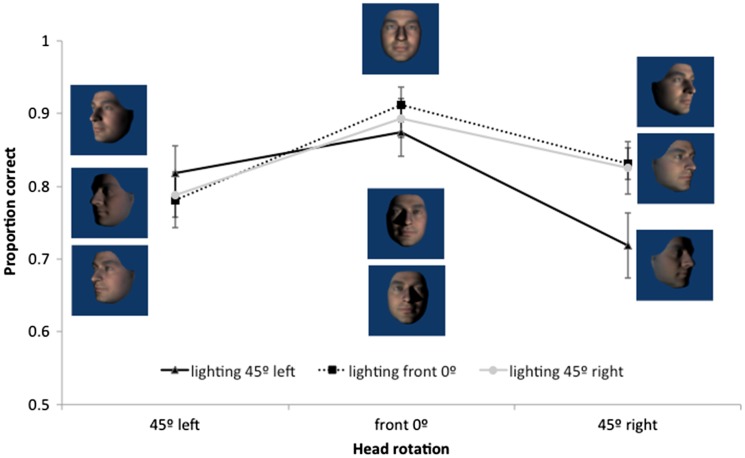
Mean proportion correct for matching upright faces following yaw head rotations of viewing and lighting angles. Error bars represent ±1 SEM.

Orientation did not interact with viewing or lighting angle but, since upright faces are of primary interest and different groups of participants were involved, we analyse and graph data for upright faces alone. As can be seen in Figure 3, participants were most accurate at matching front views and changes in yaw lighting alone had less effect on performance than changes in yaw view alone. Consistent with this a 3 (light angle) × 3 (view angle) repeated measures ANOVA for the upright face data revealed a significant effect of viewing angle, *F*(2, 30) = 8.36, *p* = .004, $\eta_{p}^{2}$ = .36, but not of lighting angle, *F*(2, 30) = 2.2, *p* = .12. Post hoc comparisons showed that front views (*M* = .89, *SD* = .08) were matched more accurately than views rotated 45° to the right (*M* = .79, *SD* = .11, *p* = .007) or left (*M* = .80, *SD* = .10, *p* < .001), but that there was no significant difference between the left and right rotated views (*p* = 1). There was no interaction between light and view, *F*(4, 60) = 2.00, *p* = .1.

#### Pitch rotations

**Figure 4.**
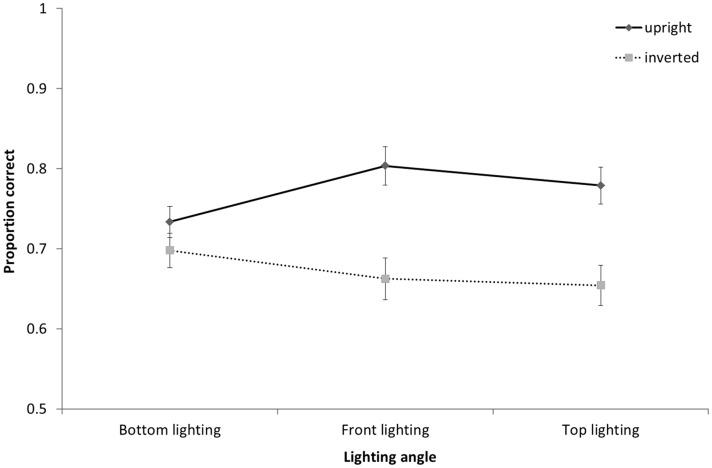
The interaction between lighting and orientation for pitch. Mean proportion correct for matching upright and inverted faces following pitch rotations of lighting angles. Note that lighting is relative to the head so that bottom lighting becomes lighting from above after inversion. Error bars represent ±1 SEM.

As with yaw rotation analysis, the three-way mixed-design ANOVA on pitch rotations showed a main effect of the between subjects factor of orientation, *F*(1, 33) = 20.48, *p* < .001, $\eta_{p}^{2}$ = .38. Matching accuracy was again significantly greater for upright (*M* = .77, *SD* = .06) than inverted (*M* = .67, *SD* = .07) faces. There was also a significant main effect of pitch view, *F*(2, 66) = 25.9, *p* < .001, $\eta_{p}^{2}$ = .44, but not lighting angle *F*(2, 66) = 0.43, *p* = .65. Orientation interacted with lighting angle, *F*(2, 66) = 3.60, *p* = .03, $\eta_{p}^{2}$ = .10 (see Figure 4). Post hoc comparisons showed that while accuracy was higher for upright than inverted faces for both front and top lighting (*p* < .001 and *p* = .001, respectively), there was no significant effect of orientation for bottom-lit faces (*p* = .23). Neither the orientation by view interaction nor the three-way interaction with orientation was significant (both *p* > .2).

**Figure 5.**
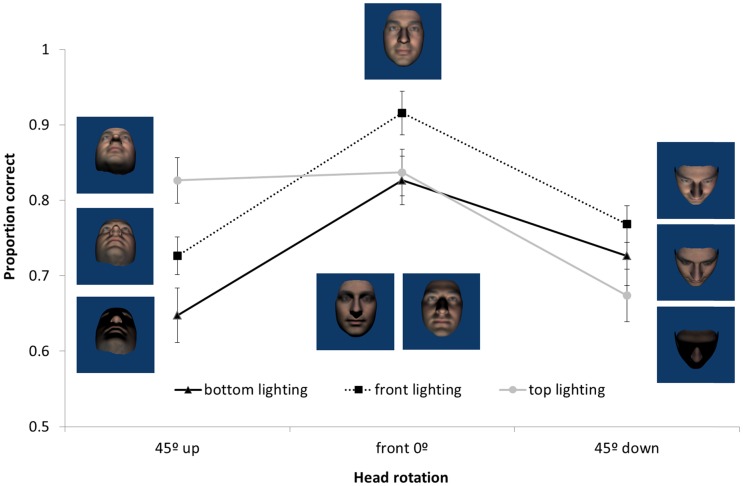
Mean proportion correct for matching upright faces following pitch head rotations of viewing and lighting angles. Error bars represent ±1 SEM.

A 3 (light angle) × 3 (view angle) repeated measures ANOVA on upright faces revealed a significant Light × View interaction, *F*(4, 72) = 5.00, *p* = .001, $\eta_{p}^{2}$ = .22, as well as significant main effects of lighting, *F*(2, 36) = 4.80, *p* = .01, $\eta_{p}^{2}$ = .21, and viewing angle, *F*(2, 36) = 14.77, *p* < .001, $\eta_{p}^{2}$ = .45. Figure 5 shows how the effect of lighting conditions depended on head rotation. Post hoc comparisons show that, when the face was rotated down (Figure 5 right), there were no significant differences between lighting conditions (all *p* > .08). When the face was viewed from the front, front lighting produces more accurate performance than either bottom (*p* = .01) or top (*p* = .05) lighting which did not differ from each other. When the face was rotated up, matching accuracy with lighting from above was significantly higher than with lighting from below (*p* = .001), but not significantly different to lighting from in front (*p* = .09). There was no significant difference between front and bottom lighting (*p* = .23).

**Figure 6.**
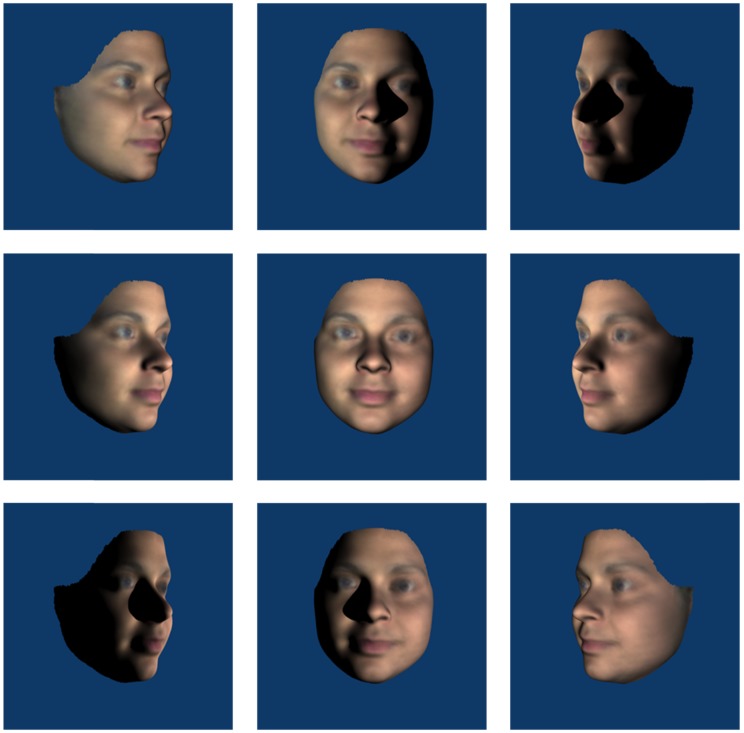
Examples of stimuli in which the *camera* was rotated about the yaw axis (left column 45° leftwards, middle column 0° and right column 45° right wards) and lighting rotated about the yaw axis (top row 45° leftwards, middle row 0° front lighting and bottom row 45° rightwards). Note that because we have now manipulated camera rotation, the layout of images does not match with those in Figure 1.

**Figure 7.**
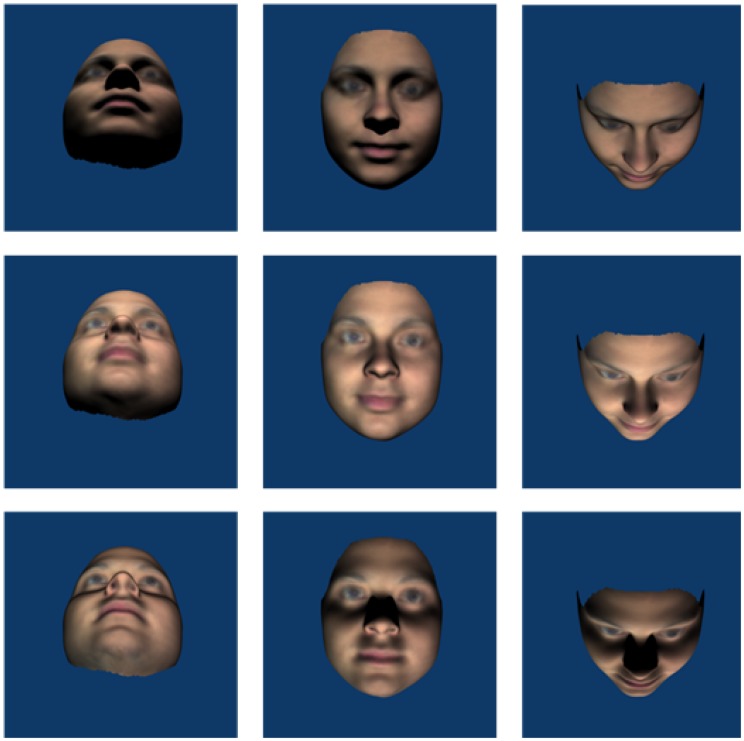
Examples of stimuli in which the *camera* was rotated about the pitch axis (left column 45° below centre, middle column 0° and right column 45° above centre) and lighting rotated about the pitch axis (top row 45° top lighting, middle row 0° front lighting and bottom row 45° bottom lighting). Note that because we have now manipulated camera rotation, the layout of images does not match with those in Figure 2.

### Discussion

We found an overall effect of inversion with greater accuracy in matching upright than inverted faces. Analyses of the yaw data showed no interactions with orientation which shows that the inversion effect was independent of changes in the yaw angles of lighting or viewpoint. If the inversion effect is taken as an indicator of visual processes specific to upright faces (e.g., Robbins & McKone, 2003; Rossion & Boremanse, 2008), then this result suggests that the effects of yaw angle changes in view or lighting are not influenced by face-specific processing. Orientation did interact with pitch rotations of lighting, but not view. Collapsing across pitch views, matching performance was uniformly poor for inverted faces in all lighting conditions. Critically, matching performance for bottom-lit upright and bottom-lit inverted faces was equally as poor. That is, pitch-rotated views of faces that were bottom lit (see bottom row of Figure 2) showed no inversion effect, while pitch-rotated views of faces that were top or front lit (top and middle rows of Figure 2) showed typical inversion effects. This indicates that findings in which bottom lighting has reduced the inversion effect for full face views (Johnston et al., 1992) extend to pitch-rotated views of faces. Together with Hill and Bruce’s (1996) finding of a disadvantage for bottom lighting on matching across yaw viewing angle changes (not tested here), this is further evidence that bottom lighting can be detrimental to face processing. Because of the factorial combination of view and lighting in the current experiment, we can show that the lighting effects for pitch rotations cannot be accounted for simply by poor visibility of surface information. While top lighting can facilitate performance for heads rotated up, there is no similar benefit of bottom lighting for heads rotated down (see Figure 5). If the effect of pitch rotations in view were determined by the features occluded this would not be expected to depend on lighting. Lighting also determines feature visibility and performance is especially poor when lighting and view rotate in opposite directions meaning that the visible surfaces do not receive direct lighting. In Experiment 2, we further investigate the dependence of view on light while no longer including conditions where lighting and viewing direction are orthogonal.

The results of Experiment 1 suggest that overall, the effects of changing view were of a similar size for yaw and pitch ($\eta_{p}^{2}$ = .36 and .45 and η^2 ^= .15 and .18, respectively), but that their relation to lighting was different. There was no effect of yaw lighting changes, while the effect of pitch rotations in lighting were significant, they were weaker than ($\eta_{p}^{2}$ = .21 and η^2 ^= .04) and depended on the view effects. Thus, the effects of rotation in view appear to be stronger than that of equivalent rotations in lighting. There were effects of view in all lighting conditions but the effect of lighting occurred only for some pitch-rotated views.

Accuracy was the highest for matching the front-lit front view face, which may not seem surprising given that the reference face was also a front-lit front view. However, this identical image benefit was not seen for the yaw trials when compared with changes in yaw lighting direction which did not significantly affect matching performance despite introducing clearly visible image differences (central column of Figure 1). Matching performance was best when lighting and view emphasised front of face information (i.e., front view under any lighting and conditions where view and light rotate in the same direction such that lighting comes directly from in front of the face). This pattern appears more consistent with involvement of surface-based coding (where surface information such as shading and shadow is represented in visual memory for faces) than image- or edge-based encoding (Tarr et al., 1998 draw similar conclusions for object recognition). We test the nature of surface-based coding further in Experiment 2, specifically, whether illumination is represented in terms of its effect on the image or whether it is represented more implicitly with respect to the surface and shape of the face. When the head is rotated relative to a stationary and uniform light source, the pattern of light and dark is not consistent with respect to the surface of the face. If consistency in the pattern of light on a surface is important for face perception, then we might expect better performance for viewpoint (as opposed to head) rotation conditions – since the light field is uniform with respect to the head and the pattern of light and dark on the surface of the face remains unchanged. To address this, we tested moving camera conditions in Experiment 2.

## Experiment 2

It is not often the case that studies explicitly state whether images used as stimuli are created by rotation of the object or by rotation of the camera. Here, we test whether it has a systematic effect on performance. In Experiment 2, we investigated the effect of viewpoint (camera as opposed to head) rotation on face matching. That is, the head remained fixed and the camera and lighting rotated about pitch and yaw axes. In addition to testing whether the consistency of the pattern of light on the surface is important for face perception, the issue of image darkness is reduced. The rotated images created for this experiment (refer to Figures 6 and 7) are lighter compared with those in Experiment 1 (see Figures 1 and 2) and any influence of image darkness per se may be revealed in cross experiment comparisons.^2^

### Method

#### Participants

Thirty-one undergraduate psychology students from the University of Wollongong served as participants for this experiment in exchange for course credit. The approximate ratio of male to female participants was 1:4. All had normal or corrected-to-normal vision and none were familiar with the facial stimuli used in the experiment.

#### Stimuli

Same as Experiment 1 except that the camera, and not the head, was rotated to create the different viewing angle conditions. The head remained fixed (0°) with camera rotations (−45°, 0° and 45°) and lighting rotations (−45°, 0° and 45°) about the pitch and about the yaw axes. View angles were combined with lighting angles for each axis such that there were nine different views for each axis (see Figures 6 and 7). Orientation was not included as a factor in this experiment; participants only saw upright faces.

#### Procedure

The procedure was the same as for upright conditions in Experiment 1.

#### Design

We used a three-factor design: 2 Axis of Rotation (yaw/pitch) × 3 Light Angle (−45°, 0°, 45°) × 3 View Angle (−45°, 0°, 45°). The between-subject factor was axis. Light angle and view angle were manipulated within subjects. In this experiment, all faces were presented upright.

### Results and Discussion

Analyses were conducted on proportion correct face matching data. Pitch and yaw rotations were analysed separately with a 3 Lighting Angle (−45°, 0°, 45°) × 3 Viewing Angle (−45°, 0°, 45°) repeated measures ANOVA. There were 16 participants in the yaw condition and 15 in pitch.

#### Yaw rotations

**Figure 8.**
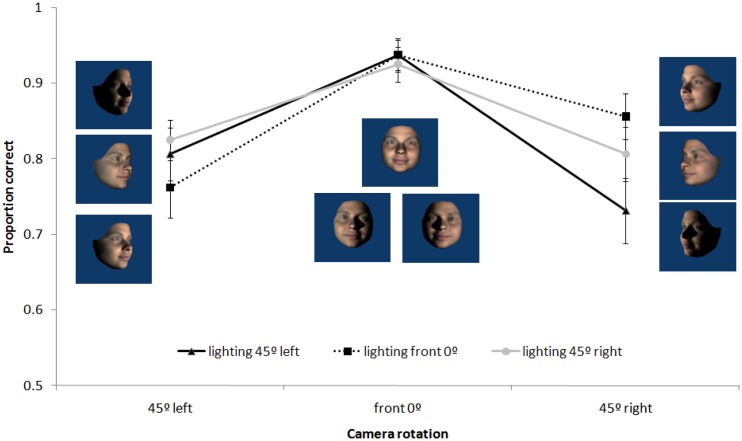
Mean proportion correct for matching upright faces following yaw rotations of camera and lighting angles. Error bars represent ±1 SEM.

A 3 × 3 repeated measures ANOVA revealed a significant interaction between yaw lighting and viewing angle, *F*(4, 60) = 3.40, *p* = .015, $\eta_{p}^{2}$ = .18. There was a significant main effect of viewing angle (*M_L__eft_* = 0.80, *SD* = 0.10; *M_F__ront_* = 0.93, *SD* = 0.06; *M_R__ight_* = 0.80, *SD* = 0.11), *F*(2, 30) = 25.40, *p* < .001, $\eta_{p}^{2}$ = .63, but no main effect of lighting, *F*(4, 60) = 0.87, *p* = .43. The interaction is illustrated in Figure 8. Superior performance in the front camera rotation condition independent of lighting is clearly visible. Post hoc comparisons show that there were no differences between lighting conditions when the camera was rotated to the left or at the front (all *p* > .38). When the camera was rotated to the right matching performance with front lighting was significantly more accurate than with left lighting (*p* = .02) but there was no difference between right and front lighting or between right and left lighting (both *p* > .43). In all three lighting conditions, front views were matched significantly more accurately than either the left or the right views (all *p* < .05). However, while there was no difference between the left and right views with left or with right lighting conditions (both *p* > .5), in the front lighting condition, right camera rotations were matched more accurately than left (*p* = .04).

#### Pitch rotations

**Figure 9.**
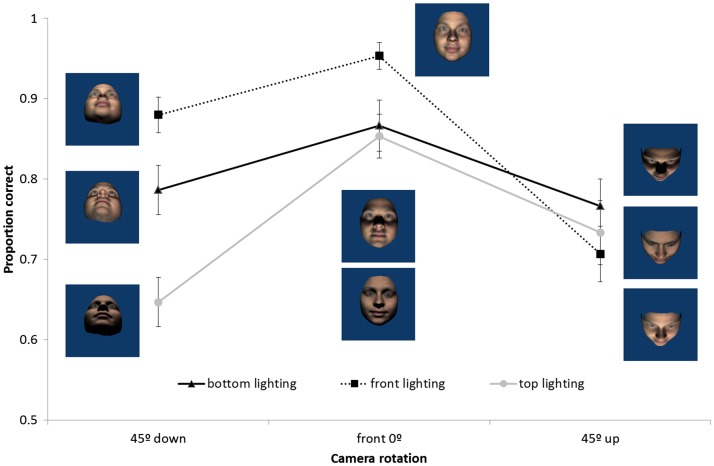
Mean proportion correct for matching upright faces following pitch rotations of camera and lighting angles. Error bars represent ±1 SEM.

A 3 × 3 repeated measures ANOVA revealed a significant interaction between pitch lighting and viewing angle, *F*(4, 56) = 5.40, *p* < .01, $\eta_{p}^{2}$ = .28. As can be seen in Figure 9, the angle of lighting had a greater effect in the camera rotated down condition than in either the camera front or camera rotated up conditions. Post hoc comparisons reveal that in the camera rotated up condition, there were no significant differences between lighting levels (all *p* > .90). In the camera front and camera rotated down conditions, matching was better for front lighting than either top or bottom lighting (all *p* < .05). Further, while a front view of the face produced most accurate performance in all lighting conditions, the patterns varied such that lighting and view appeared to be somewhat compensatory. That is, performance was partially facilitated when lighting and view were from the same direction.

#### Rotated head compared with rotated camera

Because camera rotation leaves the pattern of lighting on the surface of the face unchanged, we hypothesised that camera rotations (Experiment 2) would be less detrimental to matching performance than head rotations (Experiment 1). We tested performance across experiment groups for pitch and yaw rotations with (a) two separate mixed ANOVAs comparing equivalent image conditions and (b) independent samples *t* tests comparing unique image conditions. Of the nine image conditions created for each axis of rotation, seven are equivalent with respect to the relationship of lighting to viewpoint (see Table 1) and two are unique occurring when lighting and view are opposing (e.g., top-lit face viewed from camera rotated below).

**Table 1.** Equivalent View Pairs From Experiment 1 (Rotated Head) and Experiment 2 (Rotated Camera).

Rotated head condition (Experiment 1)	Rotated camera condition (Experiment 2)	Yaw		Pitch	
		Rotated head	Rotated camera	Rotated head	Rotated camera
Light 0°, view 0°	Light 0°, view 0°	.91 (.10)	.94 (.09)	.92 (.13)	.95 (.06)
Light −45°, view 0°	Light −45°, view 0°	.88 (.13)	.94 (.08)	.83 (.14)	.87 (.12)
Light +45°, view 0°	Light +45°, view 0°	.89 (.11)	.93 (.09)	.84 (.13)	.85 (.11)
Light +45°, view +45°	Light 0°, view −45°	.83 (.14)	.81 (.14)	.83 (.13)	.88 (.09)
Light −45°, view −45°	Light 0°, view +45°	.82 (.15)	.81 (.14)	.73 (.17)	.71 (.13)
Light 0°, view −45°	Light +45°, view +45°	.78 (.15)	.81 (.14)	.77 (.11)	.73 (.15)
Light 0°, view +45°	Light −45°, view −45°	.83 (.09)	.81 (.14)	.73 (.11)	.79 (.12)

*Note.* Mean proportion correct (*SD* in parentheses) matching for each pair in yaw and pitch rotations. Note that negative refers to left of centre or down or below and positive refers to right of centre or up or above.

Analysis of the yaw conditions in a 2 (experiment) × 7 (equivalent view type) mixed ANOVA revealed only a significant main effect of view, *F*(6, 180) = 9.13, *p* < .01, $\eta_{p}^{2}$ = .23. Neither the main effect of experiment nor the interaction was significant, both *F* < 0.8, *p* > .56. The differences between views followed the same pattern as the individual experiments where performance was best for front views regardless of lighting and poorer for left or right views. A similar analysis of the pitch conditions also revealed only a significant main effect of view, *F*(6, 192) = 13.63, *p* < .01, $\eta_{p}^{2}$ = .30 with a pattern following the individual experiments where performance was best for front view with front lighting and worst for views from above. Neither the main effect of experiment nor the interaction was significant, both *F* < 1.25, *p* > .29.

Because we cannot match the unique views across experiments, the average of these two views was compared in Bonferroni-corrected independent samples *t* tests. For yaw rotations, the matching accuracy for unique views in the rotated head experiment (*M* = .75, *SD* = .09) was not significantly different to the rotated camera experiment, *M* = .78, *SD* = .12; *t*(30) < 1, *p* = .51. Similarly, the matching accuracy of the two unique pitch views in the rotated head experiment (*M* = .66, *SD* = .10) was not significantly different than the mean accuracy of the two unique views in the rotated camera experiment, *M* = .71, *SD* = .08; *t*(32) = 1.45, *p* = .16. In summary, the comparison of performance across the two experiments showed no support of the hypothesis that camera rotations would be less detrimental to matching performance than head rotations.

## General Discussion

Regardless of whether the rotations involved the head (Experiment 1) or the camera (Experiment 2), patterns of face matching accuracy as a function of view and lighting were similar, but distinct for the yaw and pitch rotation axes. Matching faces across changes in yaw rotations of view and lighting showed strong effects of viewpoint change with little to no effect of lighting. However, in the case of pitch rotations, changes in lighting and view each impaired performance and these effects interacted. While lighting affected matching accuracy for front views and views from below, there was no effect of lighting for faces viewed from above. Specifically, we found (a) matching front views was more accurate with front lighting than top or bottom lighting and (b) matching views from below was more accurate with bottom or front lighting than with top lighting. That is, generalisation was typically better across views when the source of light was directly (or close to directly) in front of the face. A face inversion effect, where participants were poorer at matching inverted compared with upright faces, was found in Experiment 1. The inversion effect was independent of both yaw and pitch view changes and was seen across all yaw lighting angles; however, the inversion effect interacted with pitch lighting and was evident only for faces lit from above or the front.

The presence of the face inversion effect indicates face-specific processing (Robbins & McKone 2003; Rossion & Boremanse, 2008; Valentine, 1998). In this study, face inversion effects were found in matching across all pitch and yaw views and all yaw lighting conditions. These results show that matching even unfamiliar faces is not simply determined by image similarity (which does not change with orientation) and suggests a role for class based knowledge. Notably, while face inversion effects were found for top- and front-lit pitch views, no inversion effect was found for pitch view of faces with bottom lighting. As can be seen in Figure 4, bottom lighting was detrimental to matching accuracy for upright faces rather than providing an advantage to inverted faces. That is, the lack of an inversion effect in this case is a result of bottom lighting affecting upright (and not inverted) face processing. The face inversion effect has been argued to be due to an inability to process configural cues from inverted faces (Freire, Lee, & Symons, 2000; Leder & Bruce, 2000), thus, the detrimental effect of bottom lighting on face matching may also be due to a disruption to the processing of configural information cues. Bottom lighting has also been shown to disproportionately impair use of cues for 3D shape (Hill & Bruce, 1996; Johnston et al., 1992). While we cannot rule out the possibility that we may simply be more familiar or experienced at face perception in front- and top-lit conditions, together these results suggest that front and top lighting facilitates configural and 3D shape processing for faces. Subsequent discussion is restricted to upright faces.

The results of Experiment 1 showed that left and right yaw rotation of the head reduced matching performance but yaw rotation of lighting angle did not. Head rotation changes outline, aspect ratios and shape of projected features which lighting does not. Changing yaw lighting had no effect on performance despite introducing changes to the image (via light patterns on the face). However, while there were head rotation effects in pitch, changes in pitch lighting angle also affected face matching performance in a way that yaw changes did not. The bilateral symmetry of the face is clearly critical. The pattern of lighting on the lit side of the face in the case of yaw rotations supports accurate matching. There is substantial overlap between front images with yaw-rotated lighting (see centre columns of Figures 1 or 6) and the reference image where half of the yaw lit face is nearly identical to the reference image. This, plus the use of *virtual views* (Troje & Bülthoff, 1996) to compensate for information obscured by shadow, could account for the lack of lighting effect. The pattern of light resulting from rotations in pitch lighting, however, provides no overlap with the reference image (see centre columns of Figure 2 or 7) and virtual views are of no use given the bilateral symmetry of the face. A novel finding is the interaction between view and lighting effects in pitch. While there is better matching performance for front-lit front views and top-lit views of the head rotated up, there is no effect of lighting when the head is rotated down. Bottom lighting in this case results in front-of-face lighting and high surface visibility. Top lighting, although orthogonal to viewing direction, provides outline information of the nose and brows which are the more prominent features in this view. Further, no difference between top and bottom lighting rules out a general explanation of lighting effects in terms of a light-from-above assumption.

In Experiment 2, view was manipulated as a change in camera viewpoint rather than head rotation. Despite different effects on the image (e.g., the introduction of internal cast shadows and the overall *darkness*), matching performance did not vary significantly across experiments suggesting that information regarding the pattern of lighting on the face is not critical for matching. With regard to yaw rotations, again there was an effect of a change in view and no effect of a change in lighting; however, there was a significant interaction between lighting and view as a change in camera rotation. There was no effect of light for front- or left-rotated camera views, but there were differential effects of lighting for right-rotated camera views. That is, the pattern of light on the face is important for matching right-rotated camera views but not others. General surface visibility cannot account for this result since left-rotated camera views saw good matching performance even when view and lighting were orthogonal. Since this view sees the majority of the face information on the left hand side of the image (see Figure 6 right hand column), and there is evidence of a left visual field bias in face perception (e.g., Burt & Perrett, 1997; Gilbert & Balkan, 1973), it is possible that lighting had a greater effect in right-rotated camera views where face processing may be relatively dominant. This can only be a partial explanation, however, since a similar finding was not made in Experiment 1 and further research would be needed to test this idea.

The effect of changes to view and lighting in pitch was very similar for camera and head rotations (see Figures 5 and 9). Again, matching was impaired by rotating the camera view from the front view. Changes in pitch lighting alone affected matching performance (which was never found for yaw lighting) and the effects of lighting and view interacted. While matching performance was better for lighting from in front of the face for front views and views from below, there was no effect of lighting for views from above. A difference in the pattern of lighting effects between camera and head rotation on views from below suggests a role for surface information. When the head was viewed from below as a result of the head being rotated up, we found typical lighting effects where top lighting produced better performance than bottom lighting. However, when the head was viewed from below as a result of the camera being rotated down, top lighting resulted in the worst performance. Notably, it was conditions in which faces were lit from an in-front-of-face source (providing most surface information) that lead to best performance in pitch conditions.

Overall, this study shows overall stronger viewpoint effects than lighting effects in a matching task. This asymmetry reflects patterns found in face learning studies. Liu, Bhuiyan, Ward, and Sui (2009) showed that learning faces at multiple poses (yaw rotations between 0° ± 62°) resulted in better transfer to a new lighting condition (left vs. right lighting) than learning a single pose. However, no such benefit was found for learning a face in multiple lighting conditions. They concluded that while viewpoint training can compensate for lighting variation, lighting conditions may only prove useful for a trained viewpoint. That is, viewpoint processing plays a stronger role in image-invariant face recognition than does lighting. But in their study, viewpoint and lighting were manipulated only in the yaw axis. While along with this study, these findings point to viewpoint encoding as a primary mechanism in compensating for major image variations in both face matching and learning, we also show that where lighting does have an effect, the direction matters.

One limitation of this work is the use of synthetic faces as stimuli. Synthetic faces were used as they allowed us best control over the lighting and view angles as well as surface and texture information. While synthetic faces are not the same as and may be processed less efficiently than photographic images, there is evidence that they treated in a qualitatively similar manner in perceptual tasks (Balas & Pacella, 2015; Crookes et al., 2015; Matheson & McMullen, 2011). We also acknowledge that any conclusions are limited to situations in which the reference image is a front-lit front view of a face. Further research is needed to test generalisability to other reference image conditions.

This study has a focus on changes in pitch view, which despite their increasing relevance in surveillance situations are still much less studied than changes in yaw. Previous research has shown that face recognition is poorest in pitch views from above compared with pitch views from below and that both of these are poorer than yaw or roll views (Favelle et al., 2007, 2011), but these studies used ideal lighting (top and ambient front lighting) that may have disadvantaged recognition from pitch viewpoints. The results reported here show that the effects of pitch view rotation do depend on light. Specifically, matching pitch views from below may be facilitated by lighting that is at least approximately in front of the face, whereas there are no lighting effects when matching pitch views from above. The strength of this study is in the factorial combination of lighting and viewpoint angle in yaw and pitch separately. By making the same geometric manipulations to lighting and viewpoint in yaw as in pitch, we have shown that even though the only difference between the images in yaw and pitch groups is direction (up or down rotation vs. left or right) there are clear differences in the pattern of matching performance. Finally, a significant aspect of this study is the comparison of head versus camera rotation in creating face images, a factor not previously directly addressed in research. We show that lighting cues are exploited equally well regardless of whether the light field is uniform with respect to the surfaces of the head (camera rotation) or the pattern of light and dark changes across the surface of the face (head rotation). While clear indication of the nature of the rotation in describing methods is ideal, our results suggest that it is not likely to systematically affect results.

### Conclusions

Viewpoint and illumination change represent an important challenge for any visual system, human or computer algorithm, functioning in real-world conditions (O’Toole, 2005). Despite apparent similarities in the way that lighting and viewpoint changes affect an image, we have shown that there are clear differences in the way that these image factors affect face matching performance and that the axis of rotation matters. In line with previous research, we show that face perception is viewpoint dependent across both pitch and yaw axes. Critically, however, the effects of viewpoint changes in pitch are dependent on top or bottom lighting. Yaw (left or right rotations) manipulations of lighting angle had no significant effect alone and minimal effect on viewpoint dependence, despite surface pattern changes being just as large as for pitch manipulations of lighting angles. Thus, we can conclude that lighting effects are not being treated as a simple change in reflectance in face perception; image and edge differences cannot easily account for these findings. Rather, face perception appears to rely on representation of surface shape information and is particularly sensitive to the presence of top lighting, and not yaw lighting.

## Notes

1 The 2AFC task is associated with minimal bias. Also note that for both experiments, signal detection analysis (using a logistic measure of sensitivity, d_L_ and bias C_L_; Snodgrass & Corwin, 1988) revealed similar patterns of performance to those found for proportion correct.

2 Thanks to an anonymous reviewer for raising this issue.
